# Transmembrane BAX inhibitor motif containing 6 suppresses presenilin-2 to preserve mitochondrial integrity after myocardial ischemia-reperfusion injury

**DOI:** 10.7150/ijbs.81100

**Published:** 2023-02-13

**Authors:** Li Ma, Lihan Liao, Na Zhou, Huikang Tao, Hao Zhou, Ying Tan, Weidan Chen, Fan Cao, Xinxin Chen

**Affiliations:** 1 Guangdong Provincial Key Laboratory of Research in Structural Birth Defect Disease, Heart Center, Guangzhou Women and Children's Medical Center, Guangzhou Medical University, Guangzhou, China; 2 Department of Cardiology, Chinese PLA General Hospital, Medical School of Chinese PLA, Beijing, 100037, China

**Keywords:** TMBIM6, PS2, mitochondria, cardiac ischemia/reperfusion injury

## Abstract

Myocardial ischemia-reperfusion (I/R) damage is characterized by mitochondrial damage in cardiomyocytes. Transmembrane BAX inhibitor motif containing 6 (TMBIM6) and presenilin-2 (PS2) participate in multiple mitochondrial pathways; thus, we investigated the impact of these proteins on mitochondrial homeostasis during an acute reperfusion injury. Myocardial post-ischemic reperfusion stress impaired myocardial function, induced structural abnormalities and promoted cardiomyocyte death by disrupting the mitochondrial integrity in wild-type mice, but not in TMBIM6 transgenic mice. We found that TMBIM6 bound directly to PS2 and promoted its post-transcriptional degradation. Knocking out PS2 in mice reduced I/R injury-induced cardiac dysfunction, inflammatory responses, myocardial swelling and cardiomyocyte death by improving the mitochondrial integrity. These findings demonstrate that sufficient TMBIM6 expression can prevent PS2 accumulation during cardiac I/R injury, thus suppressing reperfusion-induced mitochondrial damage. Therefore, TMBIM6 and PS2 are promising therapeutic targets for the treatment of cardiac reperfusion damage.

## Introduction

During the pathology underlying myocardial infarction, coronary artery occlusion reduces fresh blood and oxygen to the cardiac myocyte 1. Although reperfusion and revascularization are the standard treatments to reduce myocardial ischemic injury caused by myocardial infarction, reperfusion itself can damage the heart and therefore myocardial ischemia-reperfusion (I/R) damage seems to be a complication induced by revascularization treatments 2. Clinical evidence has revealed that myocardial reperfusion challenge is closely linked to the degree of perioperative complications following myocardial infarction 3. However, there are no effective approaches to alleviate the additional damage induced by myocardial revascularization stress, since the molecular pathways underlying I/R-induced myocardial dysfunction are not fully understood.

Recent observations depicted the importance of mitochondria in the pathogenesis of myocardial revascularization stress 4-6. Although mitochondria are metabolic centers that determine the rate of oxidative phosphorylation in cardiomyocytes, they also manage numerous extracellular and intercellular signals, including those involved in the inflammatory response, calcium homeostasis, metabolic reprogramming, autophagy, oxidative stress, endoplasmic reticulum (ER) function and cell death 7-12. Mild mitochondrial injury promotes oxidative stress and impairs adenosine triphosphate (ATP) metabolism, thus reducing the contraction/relaxation capacities of cardiomyocytes 13-15. Severe mitochondrial dysfunction leads to cardiomyocyte death, followed by pro-inflammatory cell recruitment and abnormal inflammatory response activation 16, 17. Therefore, mitochondria have been regarded as potential drug targets during cardiac I/R injury.

Presenilin-2 (PS2) is a component of the γ-secretase complex, which was originally reported to cleave amyloid precursor protein. Now, ample evidence showed that PS2 is linked to Alzheimer's disease 18, and mutations in *PS2* are considered to be reliable genetic markers of Alzheimer's disease 19. Recent studies have also indicated that PS2 disrupts mitochondrial homeostasis by altering mitochondrial calcium input 20, mitochondria-ER coupling 21, mitochondrial phenotypes 22, the mitochondrial oxidative capacity 23, mitochondrial oxygen consumption, the mitochondrial membrane potential 24 and mitochondria-induced cell death 25. More importantly, mutations in *PS2* have been linked with the development of dilated cardiomyopathy and heart failure 26. PS2 protein expression was found to increase significantly in low-glucose- or hypoxia-treated cardiomyocytes 27, and knocking out PS2 was reported to increase cardiomyocyte contraction by enhancing the peak amplitudes of calcium transients 28. However, the influence of PS2 on myocardial revascularization stress has not been determined.

Transmembrane BAX inhibitor motif containing 6 (TMBIM6) is a calcium channel-like protein that is primarily localized on the surface of the ER 29. TMBIM6 is also termed as Bax inhibitor-1, since it was originally found to prevent Bax-induced mitochondrial membrane hyper-permeability and apoptosis 30. TMBIM6 has subsequently been reported to influence mitochondrial bioenergetics 31, the mPTP opening rate 32 and mitochondrial morphology 33. Genetic overexpression of *TMBIM6* was recently shown to reduce myocardial revascularization stress by preserving the mitochondrial integrity 34, although the downstream effectors of TMBIM6 in cardiomyocytes have not been defined. In this study, we investigated whether PS2 is a downstream signal of TMBIM6 and thus disturbs the mitochondrial integrity in the setting of myocardial revascularization stress.

## Materials and Methods

### Animals and I/R model

TMBIM6 transgenic (*TMBIM6^Tg^*) mice and PS2 knockout (*PS2^KO^*) mice (Jackson Laboratory) were genotyped via PCR analysis of mouse tail DNA. For the I/R experiments, ischemia was achieved through occlusion of the LAD through a 7.0 silk suture for 45 min. Then, removal of the silk suture and then restore the fresh blood of LAD to induce the reperfusion for 4 hrs, while the re-opened ligature was left in place to facilitate future analysis of the infarcted tissue 35. Mice treated with the same procedures without LAD occlusion and reperfusion were used as the sham group. TTC staining was performed to assess the infarcted area. Specifically, the mouse was anesthetized, the chest was re-opened, the LAD was re-occluded to promote the TTC perfusion into the myocardium 36. During contraction, the dye circulated and was distributed through the heart. Then, we isolated the heart which was following rinsed with cold PBS, and sliced at 1-mm intervals. Afterwards, the sections were incubated in a 1% TTC (Sigma-Aldrich) solution to visualize the infarcts and viable myocardium 37. The nonischemic area and infarcted area, were determined using computerized planimetry. These areas were comprehensively analyzed in serial sections from each mouse using ImageJ software. Mice that died within 24 hours of surgery were treated as technical errors and excluded from subsequent analyses. Mice in which the open ligatures were lost after the end of reperfusion were also excluded to ensure the accuracy of the TTC measurements 38.

### Histology

The hearts of the mice were placed in Hank's balanced solution, and then treated with 4% paraformaldehyde. Subsequently, the samples were dehydrated and treated with paraffin. Finally, an 8-µm sections of heart tissue were generated through a Microtome 6. Subsequently, HE staining was used to stain these heart sections to further observe the myocardial fiber in the presence of reperfusion stress according to a standard protocol. and then were mounted. For each group, a representative image was selected to show the average or median level of the group based on histological features and observed under a Hamamatsu NanoZoomer 2.0-HT Slide Scanner 39.

### Immunofluorescence

Mouse hearts were placed in Hanks' balanced solution and then treated with 4% paraformaldehyde. Subsequently, samples were saturated in 5, 10, 15 and 20% sucrose in PBS. Then, a 6-µm sections of heart tissues were generated through the Leica CM 3050S Cryostat (Leica Microsystems) 40. After blockade with 10% donkey serum, the sections were treated with primary antibodies including Gr1 (1:1000, Abcam, #ab25377), caspase-12 (1:1000, Abcam, #ab235180), caspase-9 (1:1000, Abcam, #ab222231) or PS2 (1:1000, Abcam, #ab51249) overnight at 4 °C. Subsequently, sections were treated with 0.1% PBST three times at room temperature and then reacted with secondary antibodies. To validate antibody specificity, the IgG isoform control from the same species was used instead of the primary antibody, and immunofluorescence was evaluated as described above 39. To distinguish genuine target staining from the background, secondary antibody-only controls were performed without the addition of the respective primary antibodies. Immunofluorescence pictures were observed under a Zeiss LSM 880 Airy Scan Confocal Microscope 41. The representative pictures were captured to show the average or median level of the group based on the fluorescence features.

### Cell culture and transfection

HL-1 cells were purchased from the Chinese Academy of Sciences (Shanghai, China). *In vitro*, HL-1 cells were subjected to hypoxic conditions for 45 min, followed by normal oxygen conditions for two hours, as previously described 42. Cell survival rate was determined using a CCK-8 assay based on our previous studies. HL-1 cells were transfected with siRNA to knowkout the expression of PS2 in the presence of hypoxia/reoxygenation condition using Lipofectamine 3000 (Invitrogen, Carlsbad, CA, USA). The TMBIM6 adenovirus was transfected into HL-1 cells based on a previous report 43.

### ELISA

ELISAs from MyBioSource, Inc. were applied to observe the changes of caspase-9 activities (Mouse Caspase 9 ELISA Kit, catalog #MBS451593), caspase-12 activities (Mouse Caspase-12 ELISA Kit, catalog #MBS9425959), ATP concentrations (Mouse Adenosine Triphosphate ELISA Kit, catalog #MBS724442), mitochondrial respiratory complex I activities (Mouse Mitochondrial Respiratory Chain Complex I ELISA Kit, catalog #MBS912812), mitochondrial respiratory complex II activities (Mouse Mitochondrial Respiratory Chain Complex II ELISA Kit, catalog #MBS108909) and mitochondrial respiratory complex III (Mouse Mitochondrial Respiratory Chain Complex III ELISA Kit, catalog #MBS108907). LDH content was also detected using an ELISA (CyQUANT^TM^ LDH Cytotoxicity Assay Kit, catalog #C20300, ThermoFisher) 34.

### Mitochondrial membrane potential and cellular oxidative stress

The relative immunofluorescence of a JC-1 probes (#ab113850, Abcam) was applied to analyze the mitochondrial potential, based on a previous study 40. Intracellular ROS levels were assessed with a Total Reactive Oxygen Species Assay Kit (#88-5930-74, ThermoFisher, Inc.) 41.

### mPTP opening detection and TUNEL staining

The mPTP opening rate was analyzed using an ELISA Kit (#ab239704, Abcam) 35. Cell death rate was detected using a TUNEL assay (#25879, CST) 6.

### Western blots

Proteins were extracted according to the conventional method 44. In brief, after blocked by 5% milk, the membranes were incubated overnight at 4℃ with PS2 antibody (#ab51249, Abcam). The protein signals were visualized using Tanon Image software (version 5100; Tanon, Shanghai, China). The signal intensity of the target protein was normalized against GAPDH, and then the fold change was calculated relative to the control group.

### Immunoprecipitation

For crosslinking immunoprecipitation, cells were co-treated with antibodies and dithiobis (succinimidyl propionate) for 40 min and then treated with 10% neutral buffered formalin (Sigma-Aldrich) 45. Before co-immunoprecipitation, siRNA or Ad-TMBIM6 were transfected into HL1 cells for 48 hours. The cells were then lysed in an immunoprecipitation buffer. The immunoprecipitation was then performed as described previously 45.

### RNA extraction, reverse transcription and qPCR

Total RNA was extracted using TRIzol™ reagent (Invitrogen) and reverse-transcribed using a Transcriptor First Strand cDNA Synthesis Kit (Roche Diagnostics, Risch-Rotkreuz, Switzerland) 46. Then, qPCR was conducted through the Fast Start Universal SYBR® Green Master Mix (Roche Diagnostics). The mRNA levels detected in each sample were normalized to *GAPDH* levels. The following primers were used: *TNFα* (Forward, 5'-AGATGGAGCAACCTAAGGTC-3'; Reverse, 5'-GCAGACCTCGCTGTTCTAGC-3'), *IL-6* (Forward, 5'-CAGACTCGCGCCTCTAAGGAGT-3'; Reverse, 5'-GATAGCCGATCCGTCGAA-3'), *MCP1* (Forward, 5'-GGATGGATTGCACAGCCATT-3'; Reverse, 5'-GCGCCGACTCAGAGGTGT-3').

### Statistical analysis

Data were presented as the mean ± SEM and analyzed using GraphPad Prism 9.0 software. For the analysis of *in vivo* experiments, data with sample sizes < 6 were subjected to nonparametric tests, as a normal distribution cannot be assessed accurately with a low sample size. The tests applied to assess significance are described in each figure legend, and the precise p-values of significant changes are indicated on the graphs. P<0.05 was significant.

## Results

### TMBIM6 overexpression reduces cardiomyocyte death upon H/R treatment

**Figure 1:**
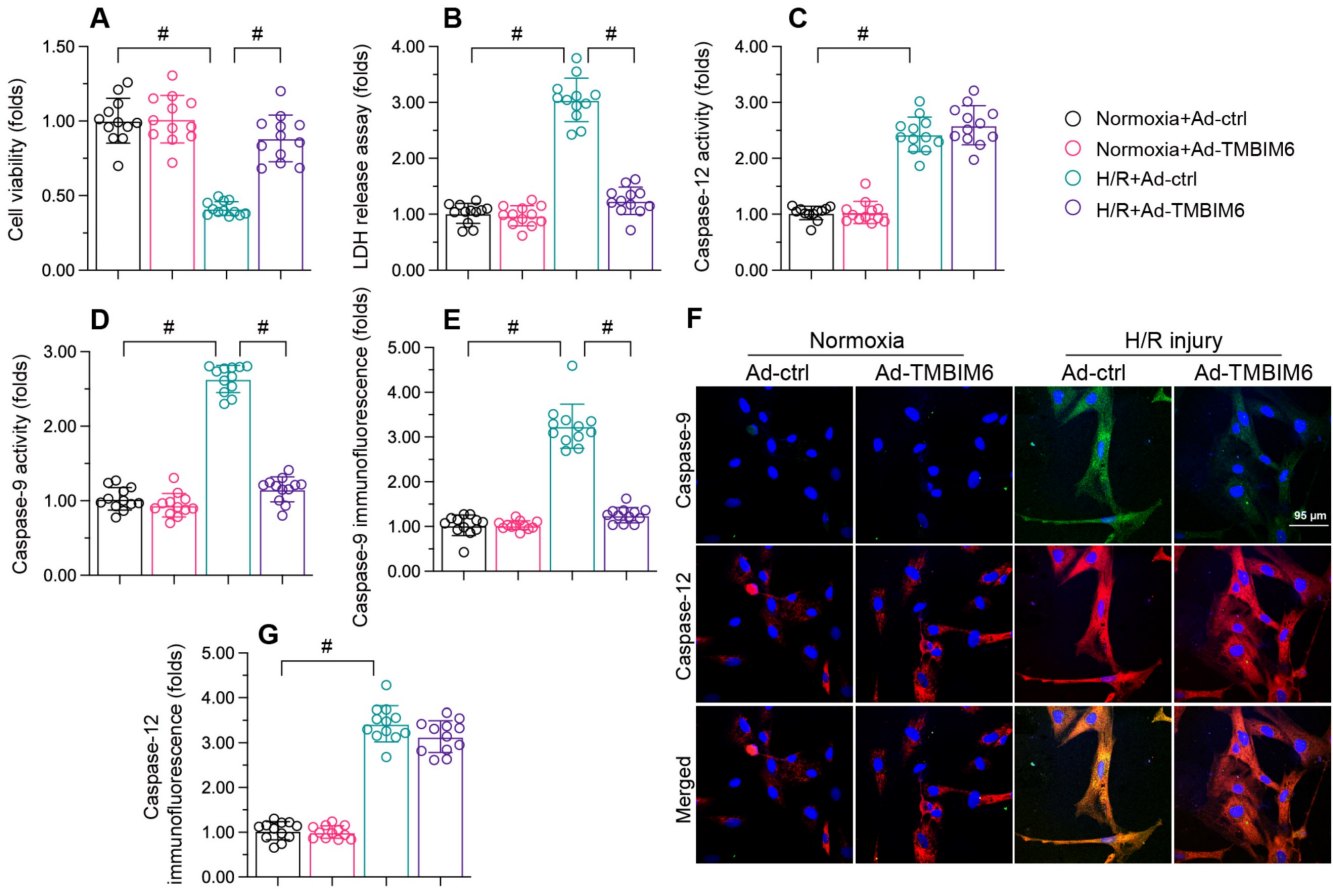
** TMBIM6 overexpression reduces cardiomyocyte death upon H/R treatment.** HL-1 cells were transfected with Ad-TMBIM6 before H/R treatment. A. Cell viability was determined with a CCK-8 assay. B. LDH release was determined with an ELISA. C, D. The activities of caspase-12 and caspase-9 were measured with ELISAs. E-G. Double immunofluorescence analysis of caspase-12 and caspase-9 in cardiomyocytes after H/R injury. #p<0.05.

To investigate the impact of TMBIM6 on cardiac reperfusion dysfunction, we transfected HL-1 cells with a TMBIM6 adenovirus (Ad-TMBIM6) and then subjected the cells to hypoxia-reoxygenation (H/R) injury. Subsequently, a CCK-8 assay showed that cellular viability was lower in the H/R injury group than in the control group (Figure 1A). Accordingly, a lactate dehydrogenase (LDH) release assay indicated that LDH release from HL-1 cells into the medium was greater in the H/R injury group than in the control group (Figure 1B). TMBIM6 overexpression protected cardiomyocyte viability (Figure 1A) and prevented LDH leakage (Figure 1B) following H/R injury.

At the molecular level, cardiomyocyte death primarily occurs via caspase-9-induced mitochondrial apoptosis or caspase-12-induced endoplasmic reticular apoptosis. To determine whether TMBIM6 overexpression improved cardiomyocyte survival by inhibiting one of these pathways, we used enzyme-linked immunosorbent assays (ELISAs) to evaluate caspase-9 and caspase-12 activity levels in HL-1 cells. The activities of caspase-9/12 were both significantly elevated upon H/R injury exposure; however, Ad-TMBIM6 transfection mainly prevented caspase-9 upregulation after H/R, suggesting that TMBIM6 inhibits mitochondrial apoptosis (Figure 1C and D).

To validate these results, we performed immunofluorescence assays. As shown in Figure 1E-G, the immunofluorescence signals of caspase-9 and caspase-12 in HL-1 cells were significantly elevated upon H/R injury. TMBIM6 overexpression reduced caspase-9 levels following H/R injury, but had no influence on caspase-12 levels (Figure 1E-G). These data confirmed that TMBIM6 suppresses mitochondria-induced cardiomyocyte death during cardiac post-ischemic dysfunction.

### TMBIM6 overexpression attenuates myocardial I/R damage

**Figure 2:**
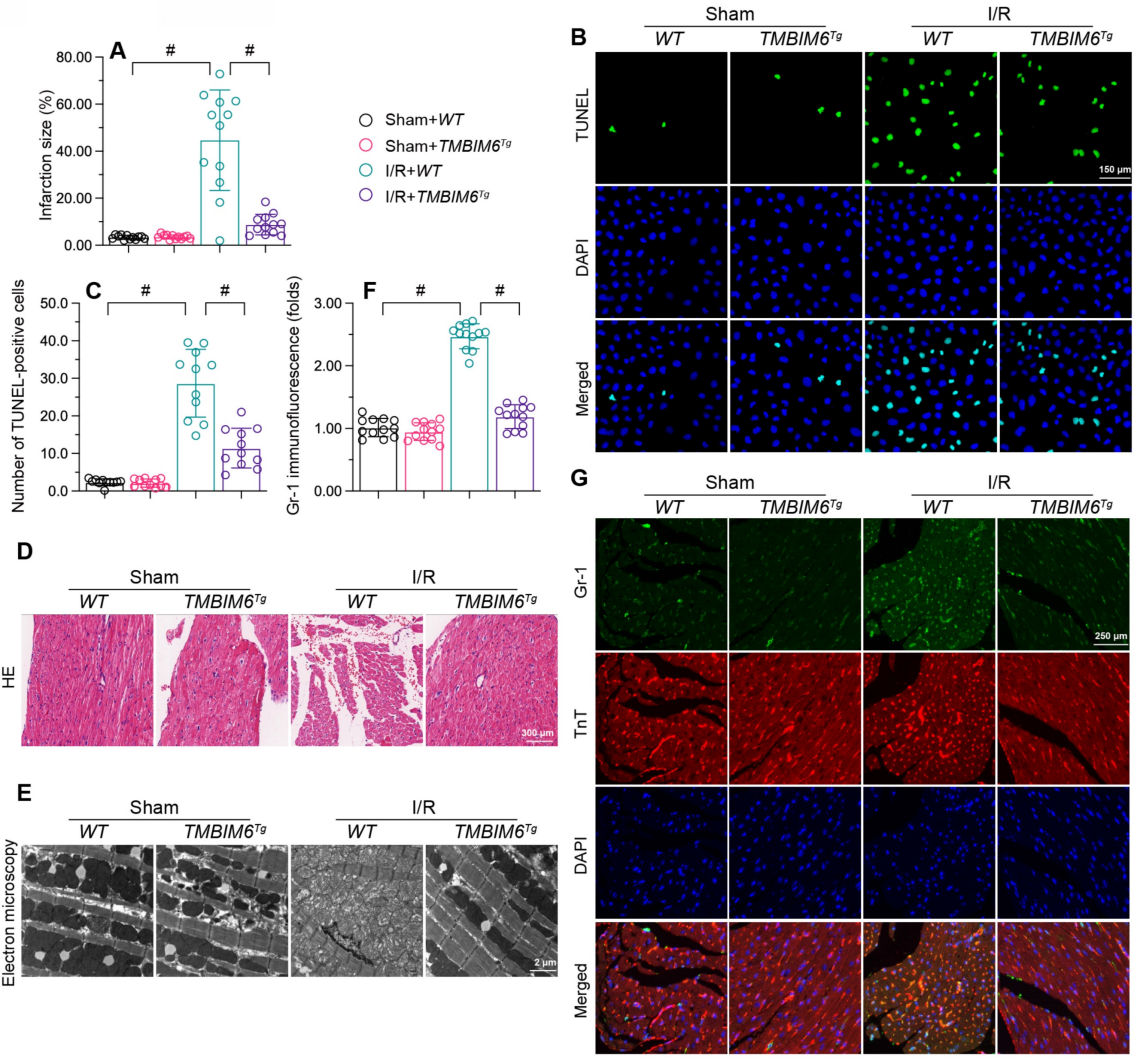
** TMBIM6 overexpression attenuates myocardial I/R injury.** *TMBIM6^Tg^* mice and *TMBIM6^flox^* control mice were subjected to cardiac I/R injury. A. Myocardial infarction size was determined by TTC staining. B-C. TUNEL staining was used to analyze the number of apoptotic cells. D. HE staining was used to analyze changes in the myocardial structure in response to I/R injury. E. Electron microscopy was used to observe ultrastructural alterations in the myocardium and mitochondria after I/R injury. F, G. A double immunofluorescence assay was used to visualize the accumulation of Gr1-positive pro-inflammatory cells within the myocardium after I/R injury. #p<0.05.

To translate our *in vitro* findings, we subjected TMBIM6 transgenic (*TMBIM6^Tg^*) or control (*TMBIM6^flox^)* mice to myocardial I/R damage or a sham operation *in vivo*. As shown in Figure 2A, I/R injury augmented the infarction area in *TMBIM6^flox^* control mice, but not in *TMBIM6^Tg^* mice. TUNEL staining showed that reperfusion elevated the ratio of dysfunctional cells in control heart tissues, but not in *TMBIM6^Tg^* heart tissues (Figure 2B and C).

Next, we used HE staining to observe changes underlying the myocardium. Compared with the sham-operated group, I/R-treated *TMBIM6^flox^* mice exhibited disorganized and swollen myocardial fibers, while this change was not observed in I/R-treated *TMBIM6^Tg^* mice (Figure 2D). We then used electron microscopy to detect ultrastructural changes in the myocardium. In *TMBIM6^flox^* control mice, I/R injury induced mitochondrial swelling and rupture (Figure 2E), followed by cytoplasmic vacuolization; however, these structural alterations were not noted in I/R-treated *TMBIM6^Tg^* mice.

Excessive cardiomyocyte death and myocardial fiber swelling can induce a cardiac inflammatory response; thus, we used immunofluorescence to evaluate inflammatory cell accumulation in the myocardium. I/R promoted Gr1-positive cells recruitment within the myocardium in *TMBIM6^flox^* mice, but not in *TMBIM6^Tg^* mice (Figure 2F and G). These results indicated that TMBIM6 overexpression reduced cardiomyocyte death, improved the myocardial structure and repressed the cardiac inflammatory response during I/R injury.

### TMBIM6 overexpression maintains heart function

**Figure 3:**
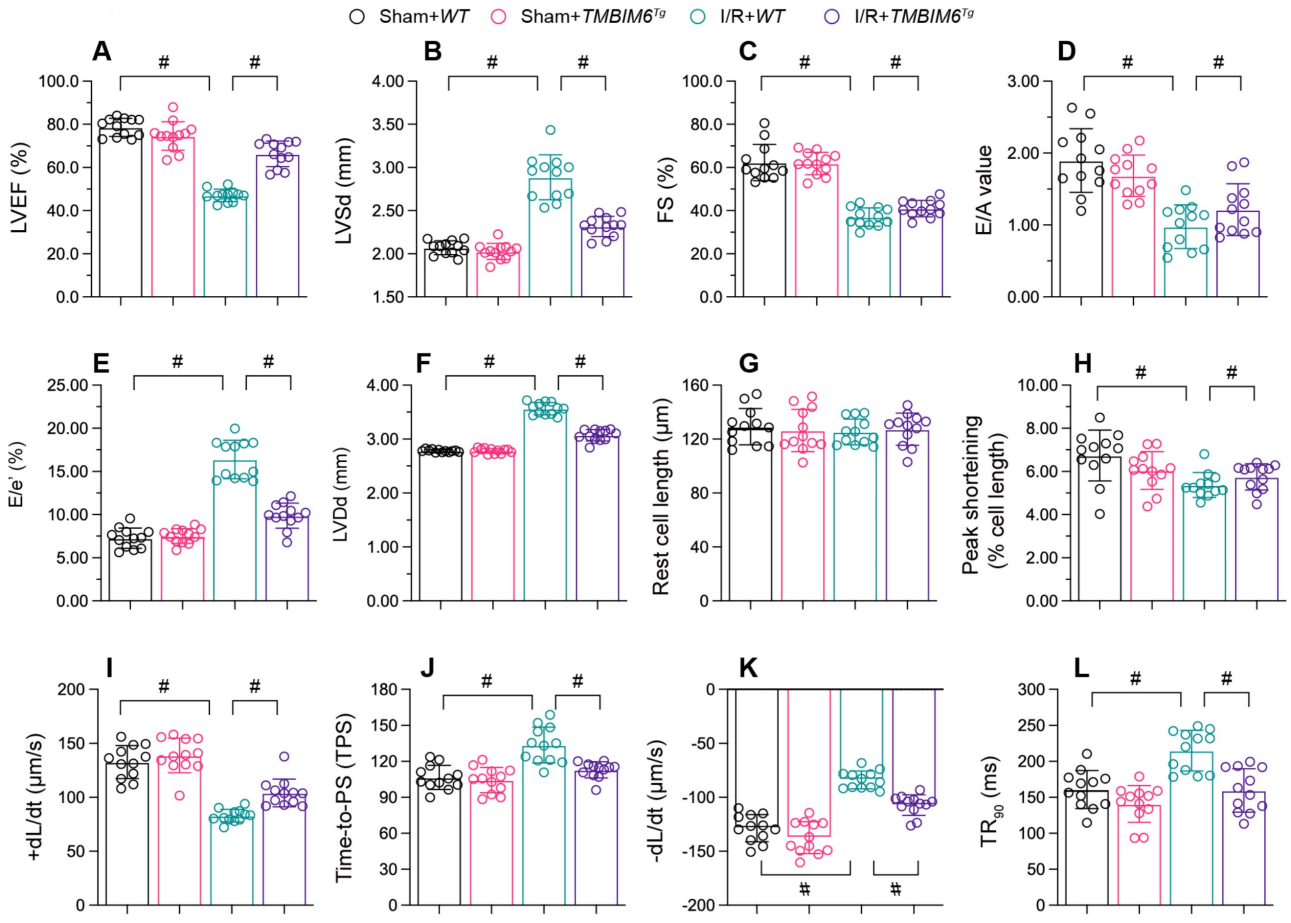
** TMBIM6 overexpression maintains heart function.** *TMBIM6^Tg^* mice and *TMBIM6^flox^* control mice were subjected to cardiac I/R injury. A-F. Echocardiography was used to assess cardiac function after I/R injury. G-L. Cardiomyocytes were isolated from the mice, and their contraction properties were analyzed. #p<0.05.

Myocardial infarction often reduces cardiac contraction/relaxation; thus, we used echocardiography to assess cardiac function in hearts. In control *TMBIM6^flox^* mice, reperfusion disrupted the cardiac systolic capacity, as evidenced by a lower ejection fraction (EF), impaired fractional shortening (FS) and augmented left ventricular systolic dimension (LVSd) (Figure 3A-C). I/R injury also impaired the myocardial diastolic capacity in these mice, as evidenced by an increased ratio of early to late transmitral flow velocities (E/A), elevated ratio of mitral peak velocity of early filling to early diastolic mitral annular velocity (E/e**'**) and amplified left ventricular diastolic dimension (LVDd) (Figure 3D-F). However, TMBIM6 overexpression normalized the cardiac systolic and diastolic capacities following reperfusion damage (Figure 3A-F).

Besides, we conducted *ex vivo* analyses of cardiomyocytes isolated from the mice after reperfusion model. I/R injury or TMBIM6 overexpression negatively affected on the resting lengths of cardiomyocytes (Figure 3G). In cardiomyocytes from *TMBIM6^flox^* control mice, I/R injury reduced the peak shortening (PS), impaired the maximal velocity of shortening (+dL/dt), repressed the time to peak shortening (TPS), blunted the maximal velocity of relengthening (-dL/dt) and elevated the time to 90% relengthening (TR90) (Figure 3H-L). However, these functional abnormalities were alleviated in cardiomyocytes isolated from I/R-treated *TMBIM6^Tg^* mice (Figure 3H-L).

### TMBIM6 overexpression sustains the mitochondrial integrity of cardiomyocytes

**Figure 4:**
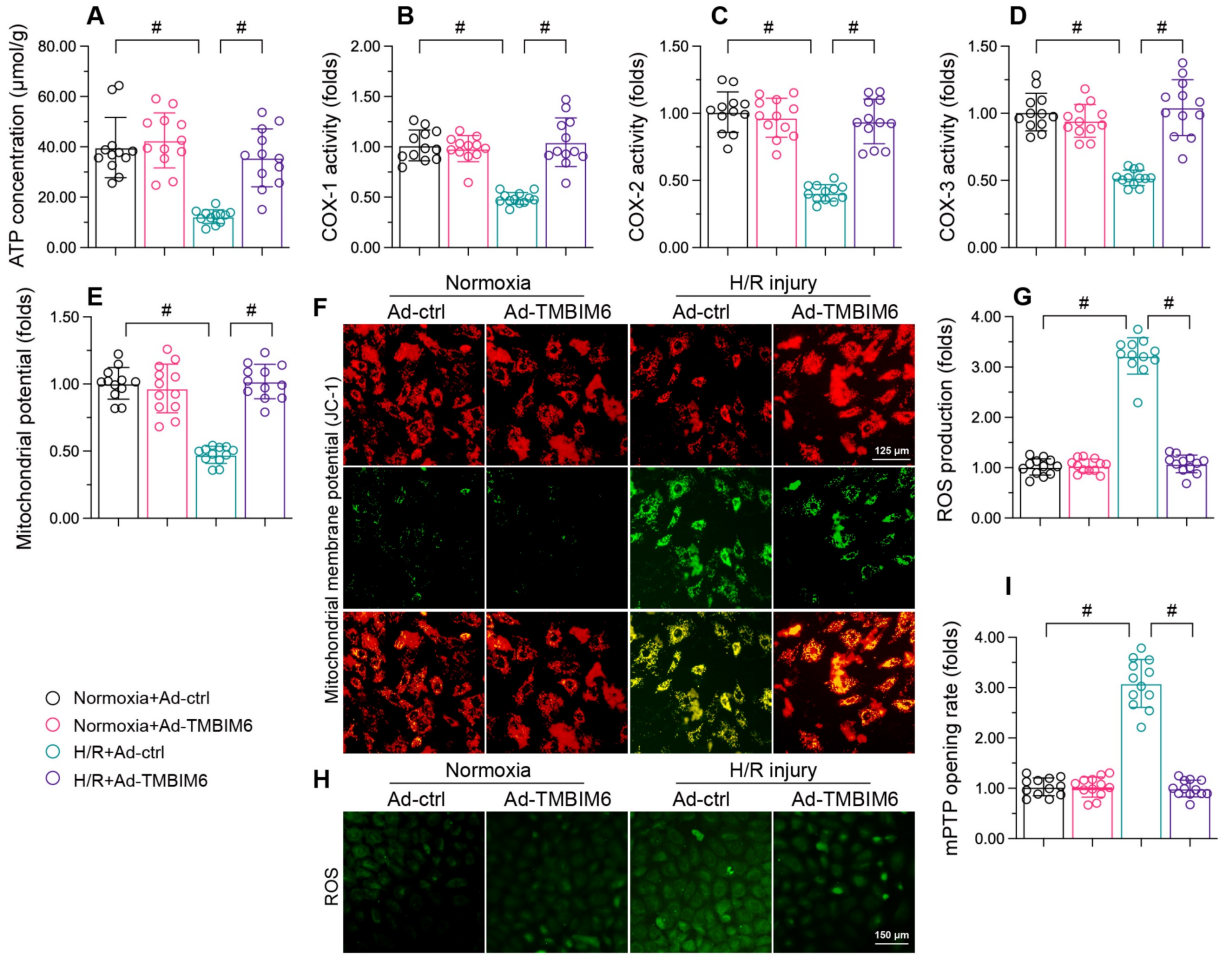
** TMBIM6 overexpression sustains the mitochondrial integrity of cardiomyocytes.** HL-1 cells were transfected with Ad-TMBIM6 before H/R treatment. A. ATP production was detected with an ELISA. B-D. The activities of the mitochondrial respiratory complexes were determined with ELISAs. E, F. The mitochondrial membrane potential was analyzed with an immunofluorescence assay using a JC-1 probe. G, H. Cellular ROS production was detected using an immunofluorescence assay in cardiomyocytes after H/R injury. I. The mPTP opening rate was recorded in cardiomyocytes transfected with Ad-TMBIM6 and subjected to H/R injury. #p<0.05.

Mitochondrial dysfunction is an important contributor to cardiac I/R injury 47-50; thus, we investigated whether TMBIM6 overexpression could normalize mitochondrial function in HL-1 cells during H/R injury. We first measured ATP production, which is primarily carried out by the mitochondrial respiratory complexes. H/R treatment reduced the ATP content in HL-1 cardiomyocytes (Figure 4A). Moreover, ELISAs indicated that H/R injury significantly repressed mitochondrial respiretory complex activity in these cells (Figure 4B-D). However, TMBIM6 overexpression maintained mitochondrial respiratory complex activity (Figure 4B-D) and therefore enhanced ATP production (Figure 4A) in HL-1 cells following H/R injury.

We then performed immunofluorescence assays, which revealed that reoxygenation stress dissipated the mitochondrial potential in HL-1 cells, whereas TMBIM6 overexpression reversed this effect (Figure 4E and F). TMBIM6 overexpression also neutralized H/R-induced cellular oxidative stress (Figure 4G and H). Moreover, H/R injury augmented the mPTP opening rate in HL-1 cells, while TMBIM6 overexpression repressed it (Figure 4I). These results demonstrated that TMBIM6 overexpression normalized mitochondrial homeostasis in the setting of cardiac revascularization stress.

### TMBIM6 binds directly to PS2 to promote its degradation

**Figure 5:**
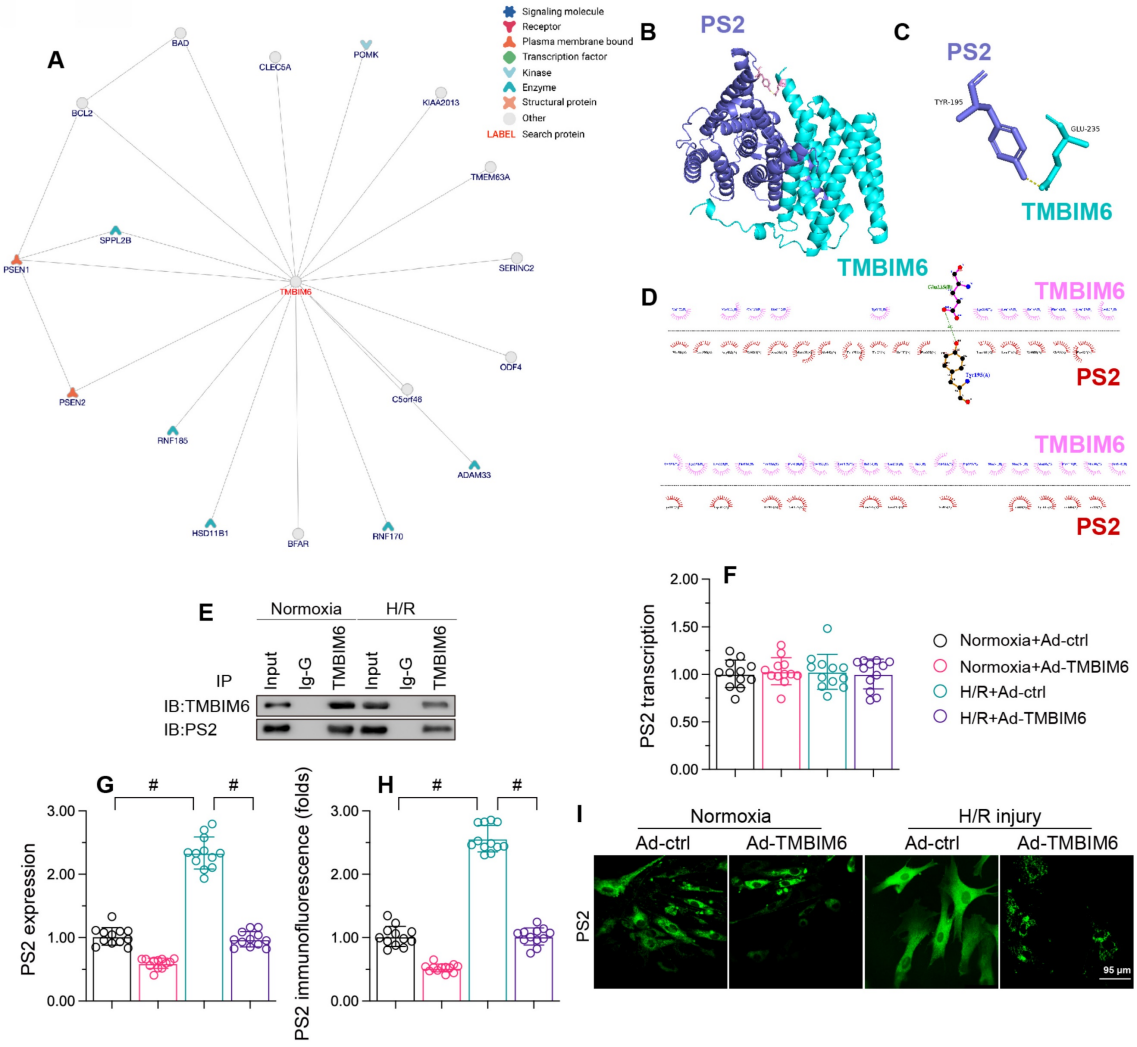
** TMBIM6 binds directly to PS2 and promotes its degradation.** HL-1 cells were transfected with Ad-TMBIM6 before H/R treatment. A. The potential protein network underlying TMBIM6 was analyzed on the inBio Discover platform. B-D. Molecular docking analysis of TMBIM6 and PS2. E. A co-immunoprecipitation assay was used to analyze the binding between TMBIM6 and PS2 following I/R injury. F. RNA was collected, and the transcription of *PS2* was analyzed via qPCR. G. Proteins were collected from cardiomyocytes, and TMBIM6 protein expression was determined via Western blotting. H, I. Immunofluorescence analysis of PS2 in cardiomyocytes exposed to H/R injury or Ad-TMBIM6. #p<0.05.

To determine the molecular mechanism whereby TMBIM6 preserved cardiomyocytes and their mitochondria function against reoxygenation stress, we focused on protein-protein interactions. First, we used the inBio Discover platform (https://inbio-discover.com) to analyze the potential protein network underlying TMBIM6 (Figure 5A). PS2, one of the potential interactive proteins, has been regarded as a regulator of mitochondrial homeostasis. Therefore, we assessed whether TMBIM6 could bind directly to PS2 to preserve mitochondrial function and integrity. A molecular docking analysis revealed several possible binding sites between TMBIM6 and PS2 (Figure 5B-D). The crosslinks between TMBIM6 and PS2 were validated through co-immunoprecipitation analyses (Figure 5E).

Next, we evaluated the effects of TMBIM6 overexpression on PS2 in HL-1 cells. Quantitative real-time PCR (qPCR) analyses demonstrated that neither reoxygenation stress nor TMBIM6 overexpression altered *PS2* transcription (Figure 5F). However, Western blotting indicated that reoxygenation stress elevated PS2 protein content, whereas TMBIM6 overexpression inhibited this effect (Figure 5G). Immunofluorescence analyses confirmed that reoxygenation stress rapidly upregulated PS2 content in HL-1 cells, whereas TMBIM6 overexpression suppressed this increase (Figure 5H and I). These results illustrated that TMBIM6 binds directly to PS2 and promotes its degradation at the protein level.

### PS2 deficiency sustains heart function during reperfusion dysfunction

**Figure 6:**
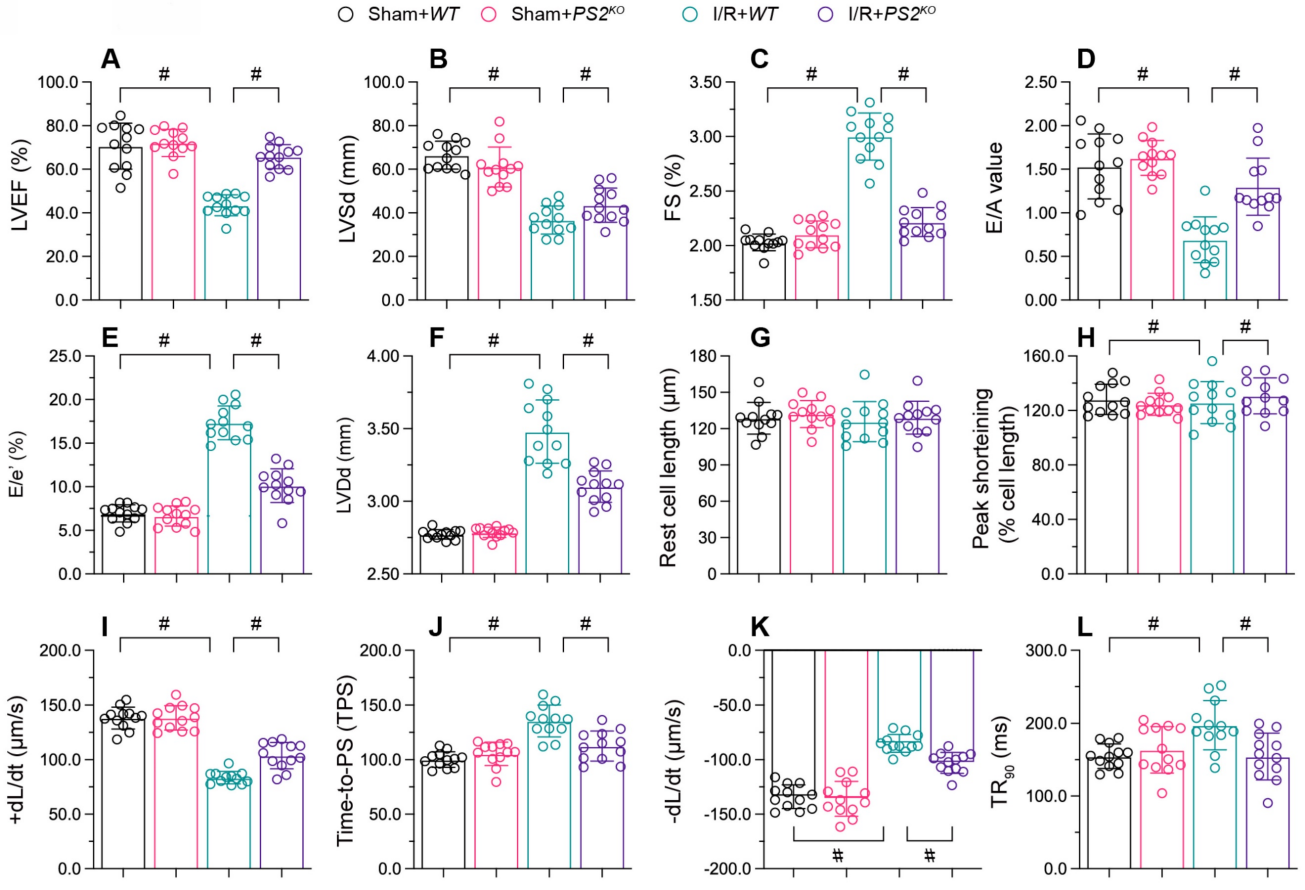
** PS2 deficiency sustains heart function during I/R injury.** *PS2^KO^* and WT mice were subjected to cardiac I/R injury. A-F. Echocardiography was used to evaluate cardiac function after I/R injury. G-L. Cardiomyocytes were isolated from the mice, and their contraction properties were analyzed. #p<0.05.

To figure out the action of PS2 on cardiac reperfusion dysfunction, we used echocardiography to compare the myocardial function of PS2 knockout (*PS2^KO^*) and wild-type (WT) mice. As shown in Figure 6A-C, I/R injury reduced the EF, suppressed the FS and augmented the LVSd in WT mice; however, these phenotypic alterations were lessened in *PS2^KO^* mice. Similarly, I/R injury impaired the diastolic capacity of the heart (E/A, E/e' and LVDd) in WT mice, but not in *PS2^KO^* mice (Figure 6D-F).

We then isolated cardiomyocytes from I/R-treated WT and *PS2^KO^* mice to evaluate their contraction properties. I/R injury blunted the PS, +dL/dt and TPS values in cardiomyocytes from WT mice, but not from *PS2^KO^* mice (Figure 6G-L). Moreover, cardiomyocytes isolated from *PS2^KO^* mice could maintain normal relaxation function after I/R injury, in contrast to those from WT mice (Figure 6G-L). These results demonstrated that knocking out PS2 normalized heart function during I/R injury.

### Knocking out PS2 attenuates I/R-induced damage in the heart

**Figure 7:**
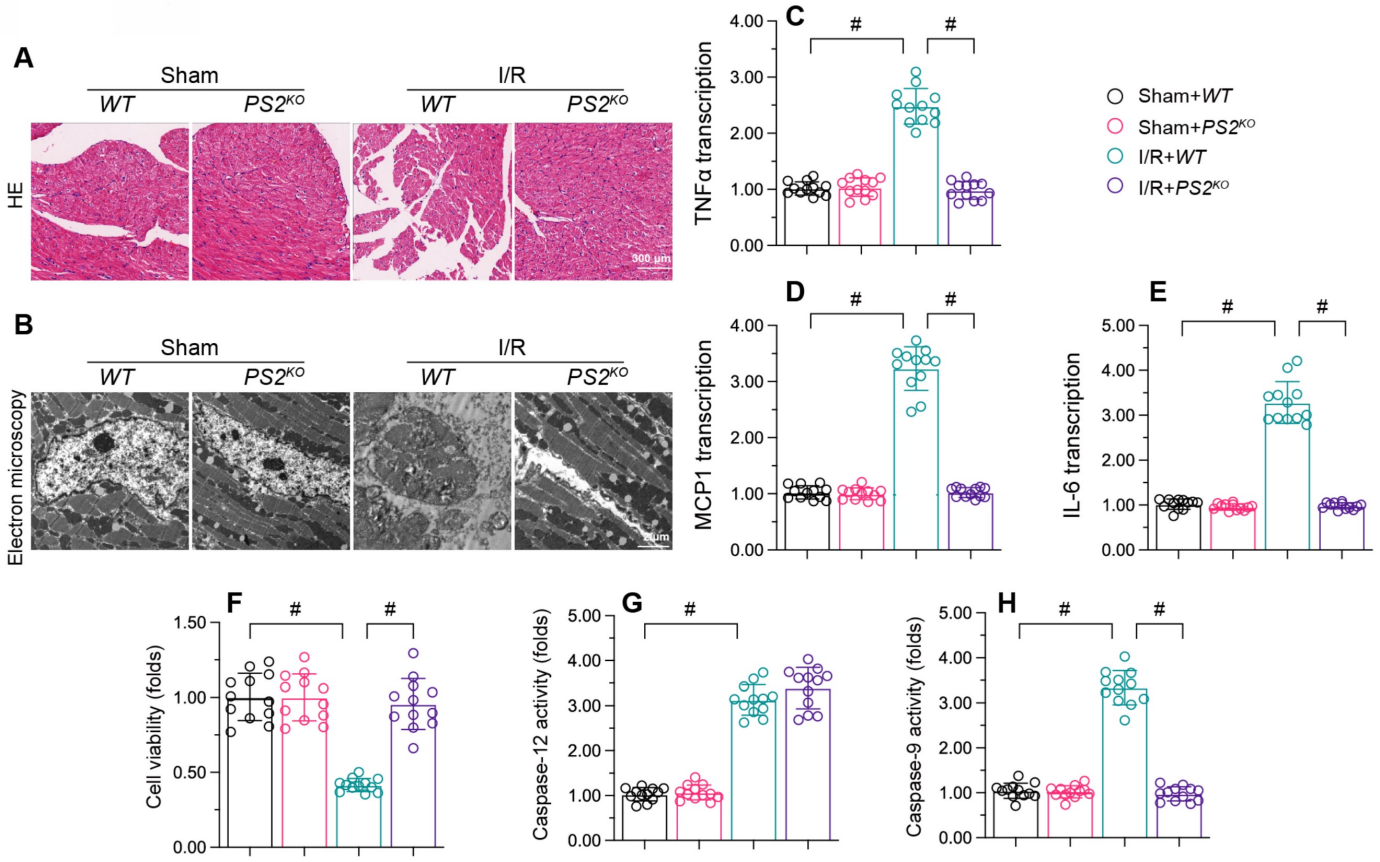
** PS2 deficiency attenuates I/R-induced damage in cardiomyocytes.** *PS2^KO^* and WT mice were subjected to cardiac I/R injury. A. HE staining was used to analyze changes in the myocardial structure following I/R injury. B. Electron microscopy was used to observe ultrastructural alterations in the myocardium and mitochondria after I/R injury. C-E. RNA was collected, and qPCR was used to analyze the transcription of *IL-6*, *MCP1* and *TNFα*. F. HL-1 cells were transfected with siRNA against *PS2*. Then, cell viability was determined with a CCK-8 assay. G, H. ELISAs were used to analyze the activities of caspase-12 and caspase-9. p<0.05.

We then assessed the effects of PS2 deficiency on cardiac damage after reperfusion dysfunction. HE assay revealed that reperfusion caused myocardial fiber swelling in WT mice, whereas this structural change was significantly ameliorated in *PS2^KO^* mice (Figure 7A). Electron microscopy indicated that I/R injury led to mitochondrial morphological disorder in myocardia from WT mice, but not from *PS2^KO^* mice (Figure 7B). Moreover, qPCR analysis of pro-inflammatory cytokines demonstrated that *IL-6*, *MCP1* and *TNFα* were significantly elevated in the myocardium following I/R injury (Figure 7C-E), while loss of PS2 prevented pro-inflammatory cytokine activation.

*In vitro*, small interfering RNA (siRNA) against *PS2* was transfected into HL-1 cells prior to H/R injury. A CCK-8 assay demonstrated that reoxygenation suppressed the viability of HL-1 cells, while PS2-siRNA negated this effect (Figure 7F). Although H/R injury promoted the activation of capsase-9 and caspase-12, PS2-siRNA treatment prevented caspase-9 activation (Figure 7G and H). These results confirmed that PS2 inhibition could alleviate I/R-induced cardiac injury.

### PS2 silencing maintains mitochondrial function in H/R-treated cardiomyocytes

**Figure 8:**
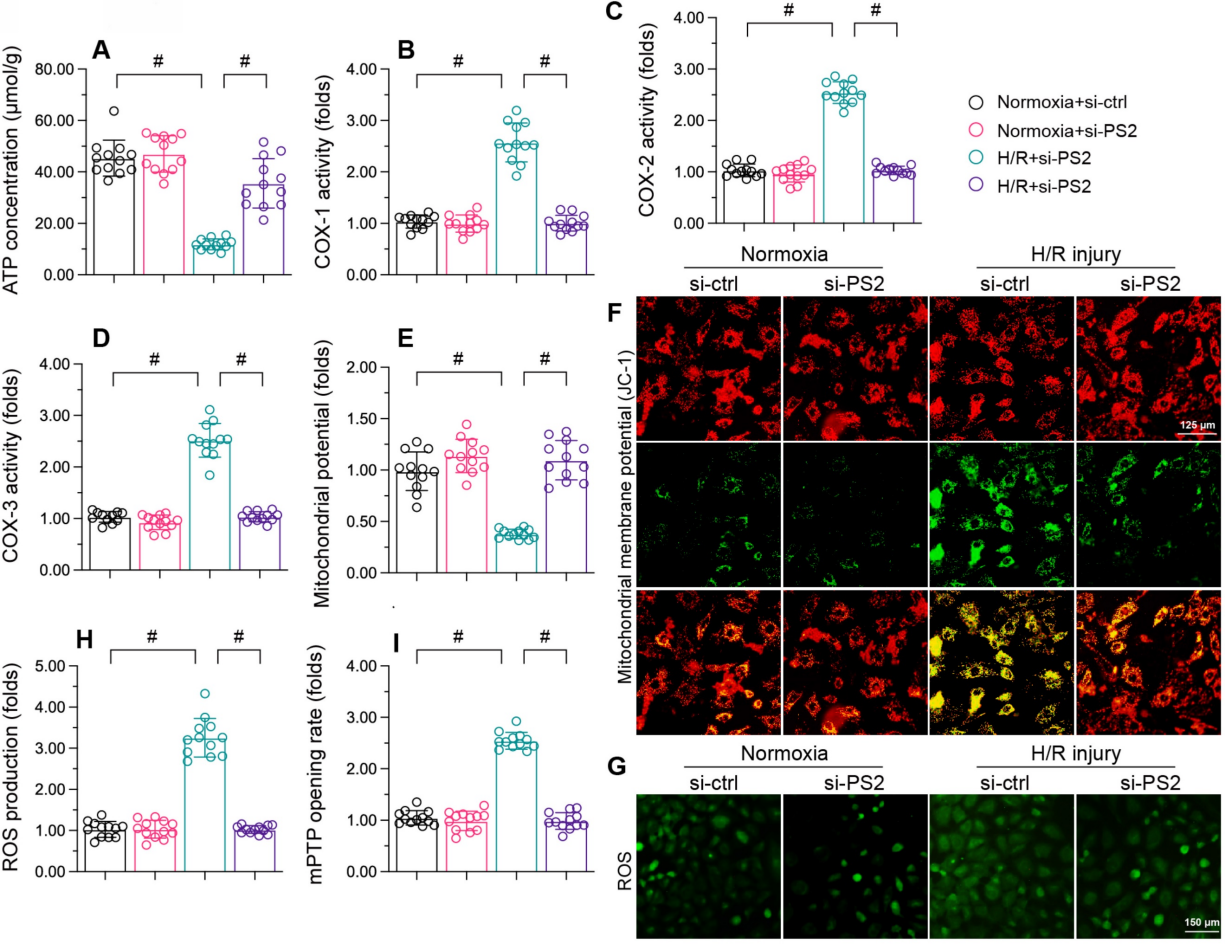
**PS2 silencing maintains mitochondrial function in H/R-treated cardiomyocytes.** HL-1 cells were transfected with PS2-siRNA before H/R treatment. A. ATP production was detected with an ELISA. B-D. The activities of the mitochondrial respiratory complexes were determined using ELISAs. E, F. The mitochondrial membrane potential was analyzed with an immunofluorescence assay using the JC-1 probe. G, H. Cellular ROS production was detected with an immunofluorescence assay in cardiomyocytes after H/R injury. I. The mPTP opening rate was recorded in cardiomyocytes transfected with PS2-siRNA and subjected to H/R injury. #p<0.05.

Finally, to determine whether PS2 inhibition protected mitochondria during cardiac I/R injury, we further analyzed mitochondrial function in HL-1 cells following PS2-siRNA treatment. As shown in Figure 8A, H/R injury repressed ATP production in cardiomyocytes, while PS2-siRNA treatment preserved ATP synthesis. PS2-siRNA also prevented mitochondrial respiratory complex inactivation (Figure 8B-D) and sustained the mitochondrial potential in reoxygenation-treated cardiomyocytes (Figure 8E and F). Moreover, PS2 inhibition markedly suppressed cellular oxidative stress (Figure 8G and H) and prevented mPTP opening (Figure 8I) following H/R treatment. Therefore, we concluded that PS2 silencing normalized mitochondrial function in H/R-treated cardiomyocytes.

## Discussion

The molecular mechanisms underlying cardiac reperfusion dysfunction were fully delineated, so there are not yet effective treatment approaches for cardiac reperfusion dysfunction in clinical practice. Our present study had three main findings: 1) TMBIM6 overexpression exerts cardioprotective effects by normalizing mitochondrial function and cardiomyocyte viability during myocardial I/R injury; 2) abnormal PS2 upregulation seems to augment reperfusion-motivated cardiac damage by disrupting the mitochondrial integrity and promoting cardiomyocyte death; 3) TMBIM6 downregulates PS2 by binding directly to it, thereby preserving the mitochondrial integrity and cardiac function. Our findings identified TMBIM6/PS2 as a novel signaling pathway in the pathogenesis of cardiac reperfusion dysfunction. Thus, stabilization of TMBIM6 expression, inhibition of PS2 activation and preservation of mitochondrial function are promising therapeutic strategies to reduce reperfusion-caused cardiomyocyte dysfunction and heart failure.

Mitochondrial dysfunction is well known to induce or exacerbate cardiac I/R injury 39, 49-52. I/R injury causes excessive mitochondrial fission, and the resulting fragmented mitochondria exhibit reduced ATP production 39, 53, 54. I/R injury also impairs mitochondrial function by inhibiting mitochondrial autophagy, thus triggering cardiomyocyte death and cardiac dysfunction 39, 47, 55-57. A reduced mitochondrial potential as well as augmented mitochondria-derived ROS generation lead to cardiomyocyte oxidative stress 58. In addition, excessive mitochondrial calcium uptake interrupts mitochondrial oxidative phosphorylation and promotes the opening of the mPTP, an early marker of cardiomyocyte necrosis 59. Therefore, mitochondria seem to be a key treatment target during myocardial reperfusion dysfunction 60. Herein, we found that reperfusion promoted mitochondrial ROS overloading, reduced the mitochondrial membrane potential, inactivated the mitochondrial respiratory complexes and suppressed ATP production. These effects worked together to induce mitochondrial dysfunction in the reperfused heart.

Our results identified PS2 as a novel inducer of mitochondrial damage, as increased PS2 expression was associated with reduced mitochondrial integrity. Accordingly, previous research described the mitochondrial involvement of PS2 in Alzheimer's Disease 61. Mutations in *PS2* were found to promote the accumulation of amyloid-β 62. PS2 deficiency was reported to induce mitochondria-ER interactions and facilitate the transfer of calcium from the mitochondria into the ER 20. Abnormal calcium signaling in mitochondria due to PS2 was found to alter the mitochondrial morphology and activate mitochondrial apoptosis 63. PS2 was also shown to enhance mitochondria-ER contact by binding to the mitochondrial fusion protein mitofusin 2 64.

In the present study, we used PS2 knockout mice to investigate the influence of PS2 on myocardial reperfusion dysfunction. We found that loss of PS2 improved the mitochondrial integrity and favored cardiomyocyte viability during I/R injury. PS2 deficiency maintained the mitochondrial potential, reduced mitochondria-derived ROS production and inhibited mitochondria-induced cardiomyocyte death, thus enhancing the viability of cardiomyocytes and elevating their resistance to reperfusion injury. To date, this is the first exploration to demonstrate that PS2 induces mitochondrial dysfunction during I/R injury.

Our results also showed that TMBIM6 is an upstream inhibitor that binds to and post-transcriptionally downregulates PS2. Through molecular docking and protein-protein interaction analyses, we demonstrated that TMBIM6 can prevent PS2 accumulation in cardiomyocytes during I/R injury. However, in our reperfusion model, TMBIM6 was markedly downregulated in the myocardium, while PS2 was upregulated. Overexpression of TMBIM6 was able to reduce PS2 expression, thereby preserving the mitochondrial integrity and enhancing cardiac function upon I/R injury.

Previous studies have had similar findings regarding TMBIM6 65. Zhou et al. reported that myocardial I/R injury suppressed TMBIM6 expression by upregulating the DNA-dependent protein kinase catalytic subunit, which recognizes double-stranded DNA damage in cardiomyocytes 34. Sufficient TMBIM6 expression was identified as a prerequisite for the activation of mitochondrial autophagy and the mitochondrial adaptive stress response, two mitochondrial quality control mechanisms that alleviate mitochondrial injury 66. Ample TMBIM6 expression was found to enhance calcium-related mitochondrial bioenergetics 31 and mitochondrial glucose metabolism 67. Moreover, TMBIM6 was shown to inhibit Bax-induced mitochondrial apoptosis 68. These results illustrate that TMBIM6 can protect the heart by attenuating mitochondrial damage and preserving myocardial function.

Overall, our data demonstrated that TMBIM6 downregulation and PS2 upregulation are pathological contributors to mitochondrial damage during cardiac I/R injury. Restoring sufficient TMBIM6 expression can prevent PS2 accumulation, thus interrupting reperfusion-induced mitochondrial damage for the benefit of the heart. Therefore, TMBIM6 and PS2 are potential therapeutic targets in patients with cardiac I/R injury.
